# Supplementary material for: Phenotypic Screen with the Human Secretome Identifies FGF16 as Inducing Proliferation of iPSC-Derived Cardiac Progenitor Cells
Source: Int J Mol Sci. 2019 Nov 30;20(23):6037. doi: 10.3390/ijms20236037 (PMC6928864; doi:10.3390/ijms20236037)
Supplement: Supplementary file 1 [file ijms-20-06037-s001.zip › ijms-613103-supplementary.pdf]

Supplementary information

Article

Phenotypic Screen with the Human Secretome Identifies FGF16 as Inducing Proliferation of iPSC-Derived Cardiac Progenitor Cells

Supplementary tables

Table S1. Excel-document. List of produced proteins tested in the hCPC screens. Column B-Uniprot annotation. Column C-gene and Column D-secreted or extracellular domain of 1-pass TM (ECD)

Table S2. Statistical significance information supporting Figures 2 and 6. EC50 values of the secretome protein hits calculated for effect on inducing hCPC, hCF and hCM, mCPC proliferation. 95% confidence intervals (CI) are indicated. Human induced pluripotent cardiac progenitor cell (hCPC), human primary cardiac fibroblast (hCF), Human induced pluripotent cardiomyocyte (hCM), mouse primary cardiac progenitor cell (mCPC). 'n/a' means the screened proteins do not have calculated EC50 values. This is either due to a small effect or no effect on cell proliferation in the screened concentration range. 'Wide' means large data variation.

Secretome Protein	Cell Type	Mean EC50 (nM)	EC50 95% Lower CI (nM)	EC50 95% Upper CI (nM)	R Squared
hFGF9	hCPC	0.69	0.22	1.52	0.90
	hCF	12.70	9.62	16.71	0.96
hFGF16	hCPC	7.46	3.72	20.40	0.91
	hCF	> 550	n/a	n/a	0.43
hNoggin	hCPC	~ 11.15	wide	wide	0.77
	hCF	n/a	n/a	n/a	n/a
hNPTX1	hCPC	~ 153.6	80.37	wide	0.72
	hCF	n/a	n/a	n/a	n/a
hVEGF-A	hCPC	~ 5.47	wide	wide	0.69
	hCF	n/a	n/a	n/a	n/a
mFGF16	mCPC	~ 8.26	wide	wide	0.91
hFGF16	mCPC	~ 1.02	wide	wide	0.84
hFGF16	hCM exp. 1	0.34	0.16	0.65	0.55
	hCM exp. 2	0.08	0.03	0.15	0.38

Table S3. Kinetic and affinity constants obtained from the kinetic evaluation of the interactions between FGF9, FGF16, hCPCs and hCFs using QCM technology. QCM measurements were performed with three replicates and data are presented as means with standard deviations.

Protein	Cell type	k_{a1} ($M^{-1} s^{-1}$)	k_{d1} ($10^{-3} s^{-1}$)	K_{D1} (nM)	B_{max1} (Hz)	k_{a2} ($M^{-1} s^{-1}$)	k_{d2} ($10^{-3} s^{-1}$)	K_{D2} (μM)	B_{max2} (Hz)
FGF9	hCPC	817 (± 31)	0.340 (± 0.05)	417 (± 57)	15.0 (± 4.0)	837 (± 31)	58.3 (± 5.0)	69.3 (± 3.1)	18.3 (± 2.5)
FGF9	hCF	1470 (± 250)	0.393 (± 0.06)	293 (± 25)	4.00 (± 0.56)	710 (± 76)	127 (± 31)	183 (± 25)	40.0 (± 2.0)
FGF16	hCPC	6830 (± 470)	3.43 (± 0.38)	507 (± 25)	28.7 (± 7.0)	14,300 (± 2500)	37.7 (± 7.6)	2.60 (± 0.40)	56.7 (± 4.5)
FGF16	hCF	Not applicable				52000 (± 3600)	49.3 (± 12)	0.967 (± 0.15)	9.00 (± 2.7)

Table S4. Receptor density of CPC and hCF on COP-1 surfaces used in the sequential addition experiments.

Protein	Cell type	
	hCPC	hCF
mFGF9	3.1×10^7	1.9×10^7
hFGF16	6.2×10^7	4.2×10^6

Supplementary Figures

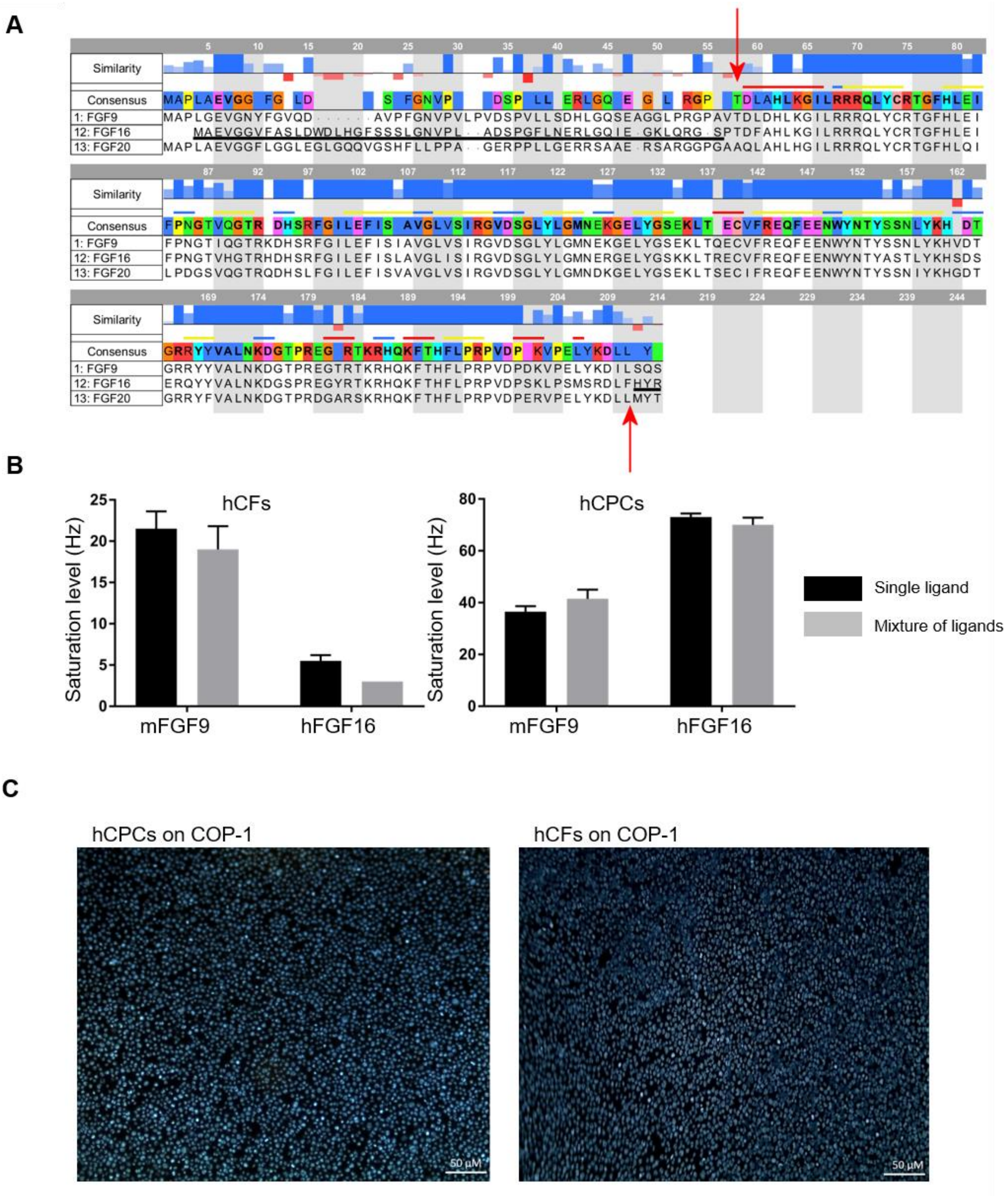

Figure S1. (**A**) Sequence alignments of human FGF9 and human FGF16. hFGF16 and hFGF9 are 73% identical in sequence. However, the conserved core has high sequence identity (84%) whereas 51 N-terminal amino acids have low sequence identity (29%). (**B**) Response level of the individual and sequential interactions of FGF9, FGF16 on hCPCs or hCFs. Left graph shows response of mFGF9 or hFGF16 injected alone or in a mixture over hCFs equilibrated with either FGF9 or FGF16. Right graph shows response of mFGF9 or hFGF16 injected alone or in a mixture over hCPCs equilibrated with either FGF9 or FGF16. The concentrations of proteins were 34 and 1 µM for m/hFGF9 and hFGF16,

respectively. The data are the mean of three individual experiments with standard deviation. (**C**) Cell coverage of hCPCs (left) or hCFs (right) on the COP-1 surface as determined by DAPI staining.

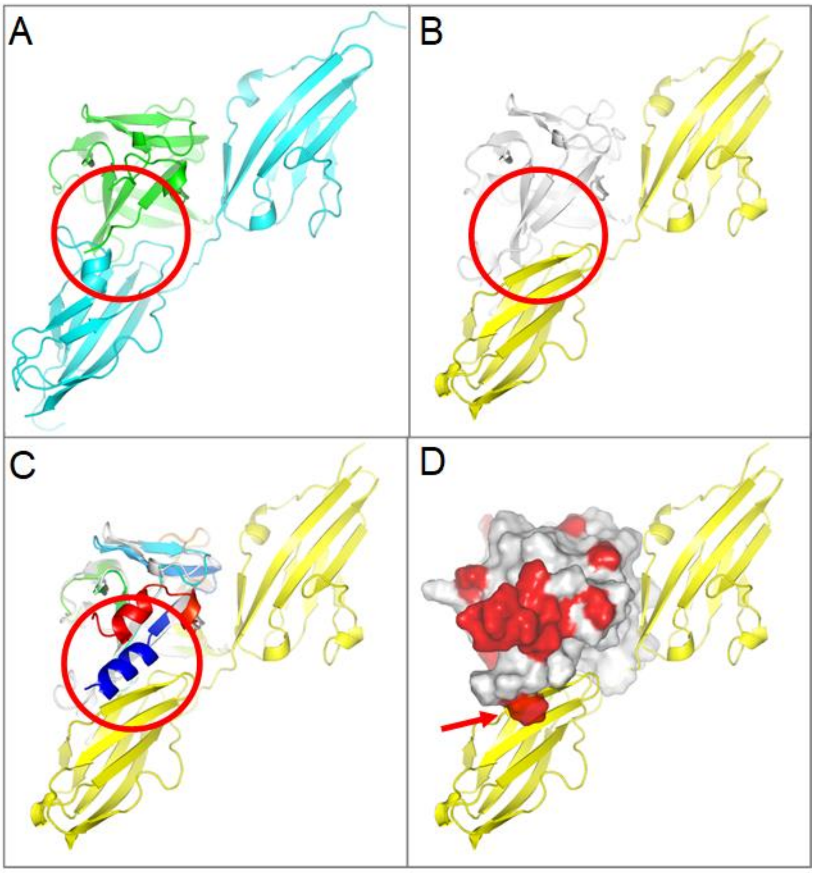

Figure S2. Structure comparison between (**A**) FGF9-FGFR1c (PDB: 5w59), (**B**) FGF8b-FGFR2c (PDB:2fdb) and (**C**) FGF16-FGFR2c (model) complexes. FGF16 homology model was generated based on X-ray structure of FGF9 (PDB:1ihk), then was superposed to FGF8b (PDB:2fdb) to form FGF16-FGFR2c model complex. Colours are used to indicate different structures, FGF9 in green, FGFR1c in cyan, FGFR2c in yellow, FGF8b in grey and FGF16 in ramped colours blue to red for N- to C-terminal. The terminal region of FGF9/16 and the βC′-βE part of FGFR1/2c are indicated in the red circles. Panel **D** shows sequence identity for the core region between FGF9 and FGF16. FGF16 model (without terminal parts) was shown as surface model with sequence differences in red. Note all the differences are away from the receptor interactions except near the N-terminus (red arrow). In the FGF9-FGFR1c complex, part of the N-terminal of FGF9 is close to βC′-βE region of FGFR1c. This part of the receptor can adapt to different FGF ligand by significant conformational changes as reported in the literature. The βC′-βE region of FGFR2c displays different secondary structures for FGF8b-FGFR2c interaction compared with that in the FGF2-FGFR2c complex [1]. It is plausible that the different terminal region of FGF16 and FGF9 can lead to different receptor interactions, respectively, similar to the observation here on FGFR2c.

Reference

1. Olsen, S.K.; Li, J.Y.; Bromleigh, C.; Eliseenkova, A.V.; Ibrahimi, O.A.; Lao, Z.; Zhang, F.; Linhardt, R.J.; Joyner, A.L.; Mohammadi, M. Structural basis by which alternative splicing modulates the organizer activity of fgf8 in the brain. *Genes Dev.* **2006**, *20*, 185–198.
